# Lobar Gross Endobronchial Disease Predicts for Overall Survival and Grade 5 Pulmonary Toxicity in Medically Inoperable Early Stage Non-Small Cell Lung Cancer Patients Treated With Stereotactic Body Radiation Therapy

**DOI:** 10.3389/fonc.2021.728519

**Published:** 2021-11-29

**Authors:** Nima Aghdam, Jonathan W. Lischalk, Monica Pernia Marin, Clare Hall, Timothy O’Connor, Lloyd Campbell, Simeng Suy, Sean P. Collins, Marc Margolis, Rebecca Krochmal, Eric Anderson, Brian T. Collins

**Affiliations:** ^1^ Department of Radiation Oncology, Beth Israel Deaconess Medical Center and Harvard Medical School, Boston, MA, United States; ^2^ Department of Radiation Oncology, Perlmutter Cancer Center New York University at Langone Hospital – Long Island, New York, NY, United States; ^3^ Geriatrics and Palliative Medicine Division, George Washington University Hospital, Washington, DC, United States; ^4^ College of Arts and Sciences, Cornell University, Ithaca, NY, United States; ^5^ Georgetown University School of Medicine, Washington, DC, United States; ^6^ Department of Radiation Medicine, MedStar Georgetown University Hospital, Washington, DC, United States; ^7^ Division of Thoracic Surgery, MedStar Georgetown University Hospital, Washington, DC, United States; ^8^ Division of Pulmonary and Critical Care Medicine, MedStar Georgetown University Hospital, Washington, DC, United States

**Keywords:** bronchoscopy, gross endobronchial disease, inoperable, non-small cell lung cancer, stereotactic body radiation therapy

## Abstract

**Purpose:**

Stereotactic body radiation therapy (SBRT) is considered standard of care for medically inoperable early stage non-small cell lung cancer (ES-NSCLC). Central tumor location is a known risk factor for severe SBRT related toxicity. Bronchoscopy allows for visualization of the central airways prior to treatment. Five fraction SBRT approaches have been advocated to mitigate treatment induced toxicity. In this report, we examine the mature clinical outcomes of a diverse cohort of ES-NSCLC patients with both peripheral and central tumors treated with a conservative 5 fraction SBRT approach and evaluate the role of lobar gross endobronchial disease (LGED) in predicting overall survival and treatment-related death.

**Methods:**

Medically inoperable biopsy-proven, lymph node-negative ES-NSCLC patients were treated with SBRT. Bronchoscopy was completed prior to treatment in all centrally located cases. The Kaplan-Meier method was used to estimate overall survival (OS), local control (LC), regional control (RC), distant metastasis free survival (DMFS) and disease-free survival (DFS). Overall survival was stratified based on clinical stage, histology, tumor location and LGED. Toxicities were scored according to the National Cancer Institute Common Terminology Criteria for Adverse Events, Version 5.0.

**Results:**

From December 2010 to December 2015, 50 consecutive patients were treated uniformly with a 50 Gy in 5 fraction SBRT approach (tumor BED_10_ ≥ 100 Gy) and followed for a minimum of 5 years or until death. At a median follow up of 42 months for all patients, 3-year OS was 50%. Three-year OS did not statistically differ between stage I and stage II disease (51% *vs*. 47%; p=0.86), adenocarcinoma and squamous cell carcinoma (50% *vs*. 45%; p=0.68), or peripheral and central tumors (56% *vs*. 45%; p=0.46). Five central tumors were found to have LGED, and 3-year OS for this cohort was quite poor at 20%. Cox regression analysis identified LGED as a predictor of OS while controlling for age, stage and location (OR:4.536, *p-value*=0.038). Despite the relatively low dose delivered, treatment likely contributed to the death of 4 patients with central tumors. Lobar gross endobronchial disease was an independent predictor for grade 5 pulmonary toxicity (n=4, p=0.007). Specifically, 3 of the 5 patients with LGED developed fatal radiation-induced bronchial stricture. Three-year LC, RC, DMFS and DFS results for the group were similar to contemporary studies at 90%, 90%, 82% and 65%.

**Conclusions:**

Central location of ES-NSCLC is a well-established predictor for severe SBRT-related toxicity. Here we identify LGED as a significant predictor of poor overall survival and grade 5 pulmonary toxicity. The relatively high rates of severe treatment-related toxicity seen in patients with central ES-NSCLC may be due in part to LGED. Underlying LGED may cause irreparable damage to the lobar airway, unmitigated by SBRT treatment thus increasing the risk of severe treatment-related toxicity. These findings should be verified in larger data sets. Future prospective central ES-NSCLC clinical trials should require staging bronchoscopy to identify LGED and further assess its clinical significance.

## Introduction

Stereotactic body radiation therapy (SBRT) is the standard of care for medically inoperable early-stage non-small cell lung cancer (ES-NSCLC). This treatment traces its roots to the early Indiana University experience by Timmerman et al., and it was in this same cohort that the authors noted a profoundly increased risk of treatment-related toxicity when an aggressive 3 fraction approach was utilized for tumors located in the center of the chest (1–4). Our understanding of this increased risk prompted investigations into safer treatment approaches to mitigate this high-grade treatment-related toxicity. Efforts to date have focused predominantly on increased fractionation as a risk mitigating approach. These efforts include those of the RTOG 0813 investigators who are currently assessing the mature outcomes of a 5-fraction SBRT approach in the treatment of central ES-NSCLC (5).

The pathophysiological etiology leading to high-grade toxicity in central lung cancers remains nebulous and is likely multifactorial in nature. It is assumed, radiation technique, total dose and fractionation schedule each play an important role in treatment-related toxicity. However, underlying pretreatment tumor-related factors likely play a significant role as well. For example, squamous cell carcinomas are more commonly seen in the central lung region and are known to have an increased risk of local failure following SBRT (6–8). It is unclear whether these tumors have an inherently more aggressive underlying biology or are associated with occult submucosal spread, both of which could lead to airway infringement and an increased risk of local failure.

Underlying tumor-related major airway erosion could be a significant, hitherto overlooked, pretreatment factor that is unmitigated by SBRT and may exacerbate treatment-related toxicity. Tumor related major airway infringement can be identified by direct visualization under bronchoscopy. Our institution routinely employs pretreatment bronchoscopic evaluation of the proximal bronchial tree in concert with staging of the mediastinum, biopsy of the primary tumor, and placement of fiducials in preparation for SBRT for the treatment of ES-NSCLC (7, 9–12). In the present study, we evaluate the role underlying pretreatment lobar gross endobronchial disease (LGED) plays in the long-term clinical outcomes of ES-NSCLC treated with SBRT.

## Materials And Methods

### Patient Eligibility

The Medstar Health Research Institute-Georgetown University Oncology institutional review board approved this study. A multidisciplinary thoracic oncology team evaluated patients. Medically inoperable patients with lymph node-negative clinical stage I or II NSCLC were included in the present analysis. Clinical staging was completed per the AJCC cancer staging manual, 7^th^ edition, which was in use during the era these patients were treated (13). Inoperable was defined as a post-bronchodilator percent predicted forced expiratory volume in one second (FEV1) of less than 50%, a carbon monoxide diffusing capacity (DLCO) of less than 50%, age greater than 75, and/or severe comorbid medical conditions. Prior to treatment, CT imaging of the chest and abdomen with IV contrast, PET imaging, and routine pulmonary function tests (PFTs) were completed when feasible. Candidates were excluded from protocol treatment if fiducials could not be safely placed for tumor tracking or if a tumor did not have pathologic confirmation prior to treatment. There was no restriction based on tumor location. A tumor was considered central if it was located within 2 cm of the proximal bronchial tree (i.e. the distal 2 cm of the trachea, primary mainstem bronchus and secondary lobar bronchi) (14). Patients with a history of chest radiation therapy were excluded from this analysis.

### Staging, Biopsy, and Fiducial Placement

**Figure 1:**
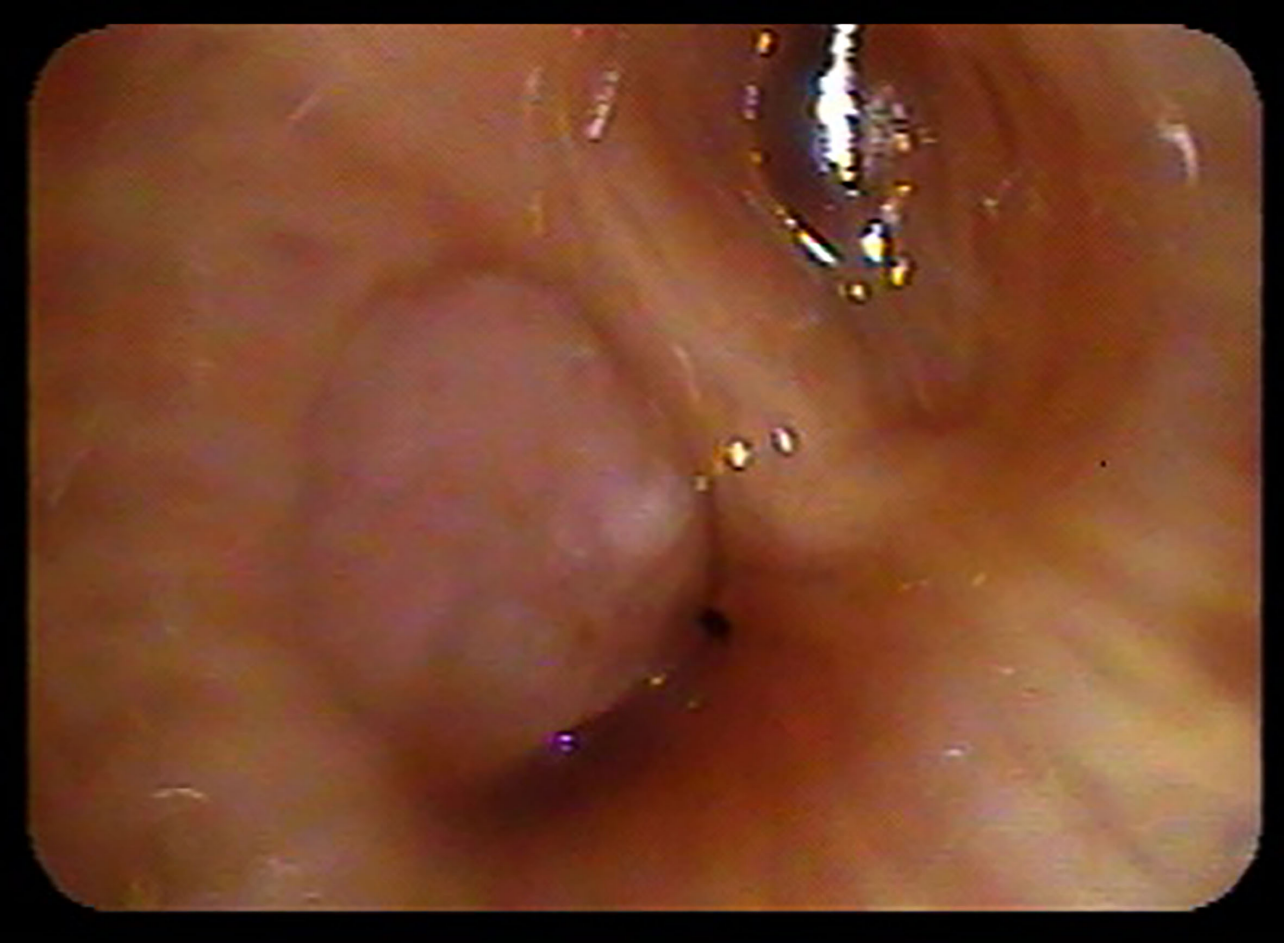
Lobar bronchus gross endobronchial disease identified during staging bronchoscopy.

Bronchoscopy was routinely completed for visualization of the central airways, mediastinal lymph node staging, primary tumor biopsy and fiducial placement as previously described (9, 15). Briefly, flexible bronchoscopy was typically performed under general anesthesia with endotracheal intubation. First, a comprehensive survey of the central airways was completed to determine if gross endobronchial disease (GED) was present (**Figure 1**). Second, endobronchial ultrasound (EBUS) was completed to determine the size of paratracheal, hilar and subcarinal lymph nodes. Next, transbronchial needle aspiration (TBNA) with three passes was completed when enlarged lymph nodes were identified (≥ 5 mm in short axis). Of note, electromagnetic navigation was frequently required to guide the bronchoscope to the tumor (15). The tumor was biopsied if pathologic confirmation of malignancy was required. Finally, 3 to 5 sterilized gold fiducials measuring 0.8-1 mm in diameter by 3-7 mm in length (Item 351-1 Best Medical International, Inc., Springfield, VA) were placed with adequate spacing (1-2 cm) in or adjacent to the primary tumor. If bronchoscopy was not feasible the same fiducials were spaced similarly under CT-guidance as previously described using conscious sedation and local anesthesia (16).

### Treatment Planning

**Figure 2:**
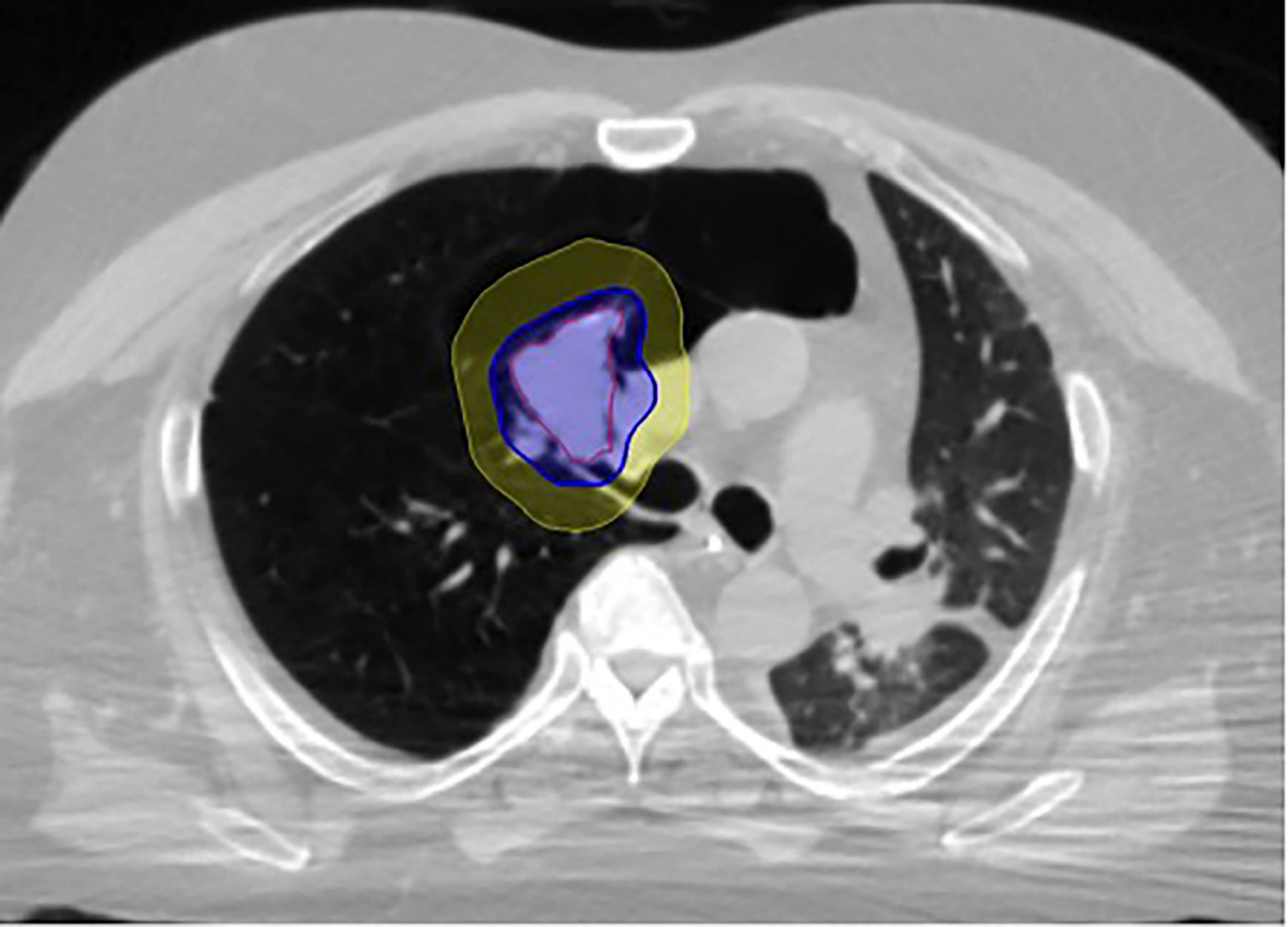
Figure 1 LGED patient SBRT treatment plan with GTV (red) and isodose color wash with 50 Gy isodose line (blue) and 35 Gy isodose line (yellow). The lobar bronchus maximum point dose in this case was 62.3 Gy.

Fine-cut (1.25-mm) treatment planning CTs were routinely obtained 7-10 days after fiducial placement during a full inhalation breath-hold. Gross tumor volumes (GTV) were contoured utilizing lung windows. The GTV margin was expanded 5 mm isotropically to establish the planning treatment volume (PTV). Routine thoracic structures were contoured and standard dosimetry completed. For central tumors, only the proximal bronchial tree (PBT) was contoured based on the RTOG atlas, and included the distal trachea, primary mainstem bronchus, and secondary lobar bronchi. Dose constraints for organs at risk were per TG-101 (17). Of note, dose constraints were not utilized for the proximal bronchial tree. A treatment plan delivering 50 Gy in five fractions (BED_10_ = 100 Gy) to an isodose line that covered at least 95% of the PTV was generated using the CyberKnife non-isocentric, inverse-planning Monte Carlo algorithm (18) (**Figure 2**).

### Treatment Delivery

Patients were treated according to the Georgetown University Hospital pulmonary nodule protocol as previously described (10). Briefly, patients were brought to the CyberKnife suite and laid supine on the treatment table with their arms at their side. Three red light-emitting diodes (LEDs) were placed on the patient’s anterior torso directed toward the camera array. Fiducials were located using orthogonal x-ray imagers. A correlation model was created between the LEDs tracked continuously by the camera array and fiducial positions imaged periodically by the x-ray targeting system. During treatment delivery the tumor position was tracked using the live camera array signal and correlation model. The linear accelerator was moved by the robotic arm to maintain precise alignment with the tumor throughout the respiratory cycle during radiation delivery. Fiducials were imaged prior to the delivery of every third beam to verify targeting accuracy and to update the correlation model.

### Follow-up Studies

Clinical evaluation and surveillance imaging were performed per routine institutional practice at 3-6 months intervals in a multidisciplinary clinic. Locoregional recurrence was defined as progression in the involved lobe or regional lymph nodes per serial PET/CT imaging as previously reported (19). Other failures were considered distant. Biopsy was required to confirm progression and direct salvage treatment. Toxicities were scored according to the National Cancer Institute Common Terminology Criteria for Adverse Events, Version 5.0 (20). Cause of death analysis was completed by two radiation oncologists (NA and BC). Autopsy was not completed to confirm cause of death.

### Statistical Analysis

Data was analyzed and graphs were prepared with the SPSS 23 statistical package (IBM Corporation, Armonk, NY). The follow-up duration was defined as the time from the date of completion of CyberKnife treatment to the last date of follow-up or the date of death. The Kaplan-Meier method was used to estimate overall survival (OS), local control (LC), regional control (RC), distant metastasis free survival (DMFS), and disease-free survival (DFS). Cox regression analysis was performed in order to identify pre-treatment variables correlating with overall survival following SBRT treatment.

## Results

### Patient, Tumor, and Treatment Characteristics

From December 2010 to December 2015, 50 inoperable, elderly (median age 75 years) and predominantly Caucasian female patients were treated (**Table 1**). Patients were followed for a minimum of 5 years or until death. Ninety-two percent of the patients were smokers with pulmonary dysfunction as the primary rationale for non-surgical treatment (**Table 2**). Thirty-one tumors were stage I (62%) with the remainder stage II. The vast majority of tumors were histologically adenocarcinoma (56%) or squamous cell carcinoma (40%). Eleven tumors were central (22%) and 39 were peripheral. The median gross tumor volume (GTV) was 31.4 cc (range 4.7 – 231.8 cc) (**Table 3**). A total dose of 50 Gy was delivered to a median 77% isodose line (range, 70% - 85%) in 5 treatments over a 5 to 10-day period (median of 7 days). The median maximum dose to the GTV was 65 Gy (rang 59-71 Gy). Median Dmax to lobar bronchi, mainstem bronchi and the trachea for central tumors was 62.3 Gy (range, 33.7-68 Gy), 30.8 Gy (15.2-59.3 Gy) and 12.1 Gy (1.1-37.1 Gy), respectively.

**Table 1:** Patient characteristics.

Age	
Median	75
Range	58-94
Sex, n (%)	
Male	23 (46)
Female	27 (54)
Race, n (%)	
Caucasian	34 (68)
African	15 (30)
Asian	1 (2)
ECOG, n (%)	
0-1	35 (70)
>=2	15 (30)

**Table 2:** Pulmonary function characteristics.

FEV1, L	
Mean	1.41
Range	0.52-2.85
% predicted FEV1	
Mean	58.55
Range	22-105
DLCO, mL/min/mmHg	
Mean	10.44
Range	5.10-16.70
% Predicted DLCO	
Mean	52.6
Range	19-102

**Table 3:** Tumor characteristics.

Clinical stage, n (%)	
Stage I	31 (62)
Stage II	19 (38)
Histology, %	
Adenocarcinoma	28 (56)
Squamous cell carcinoma	20 (40)
NSCLC NOS	2 (4)
Location, %	
Peripheral	39 (78)
Central	11 (22)
Gross Tumor Volume, CC	
Median	31.4
Range	(4.7-231.8)

### Disease Control and Survival

**Figure 3:**
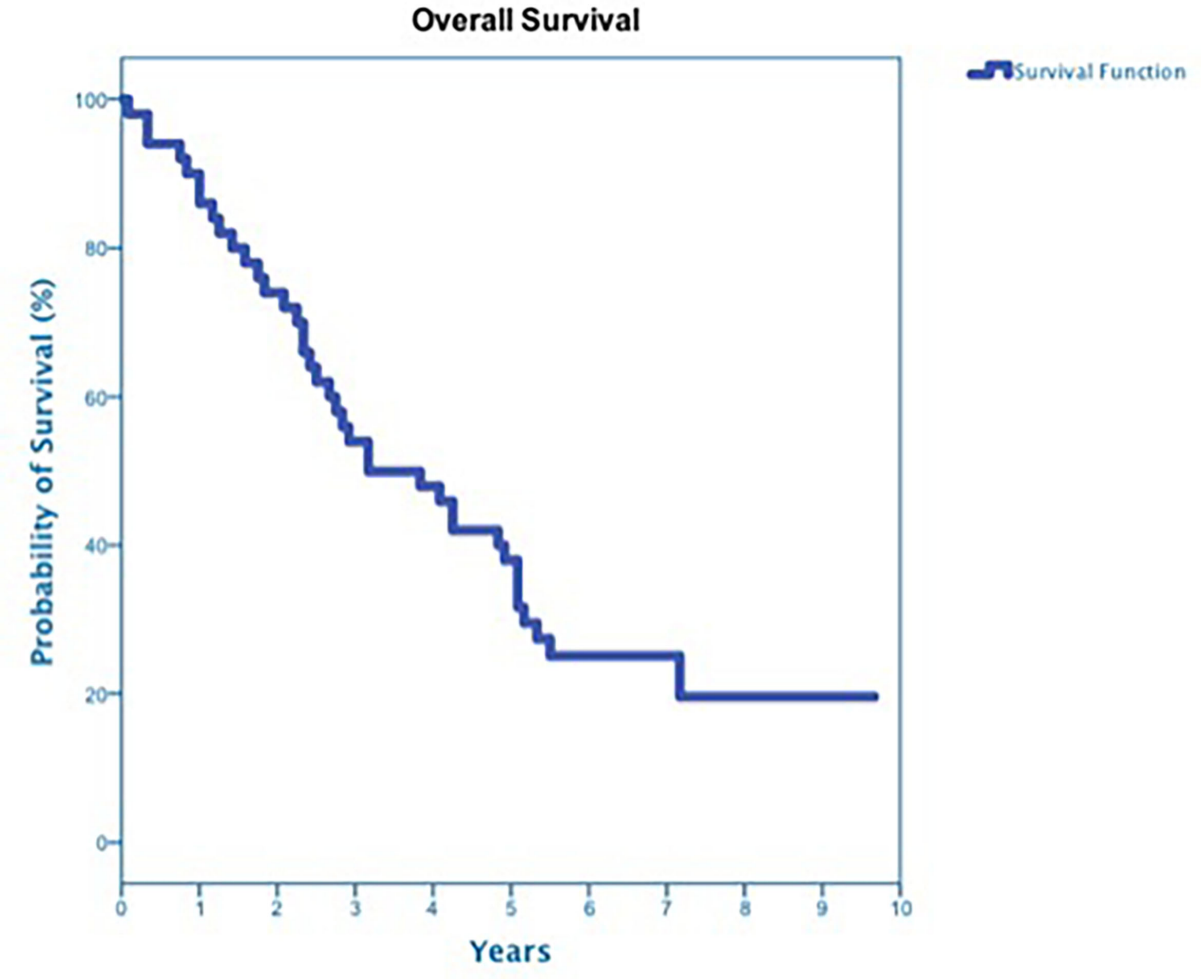
Overall Survival for All patients.

**Figure 4:**
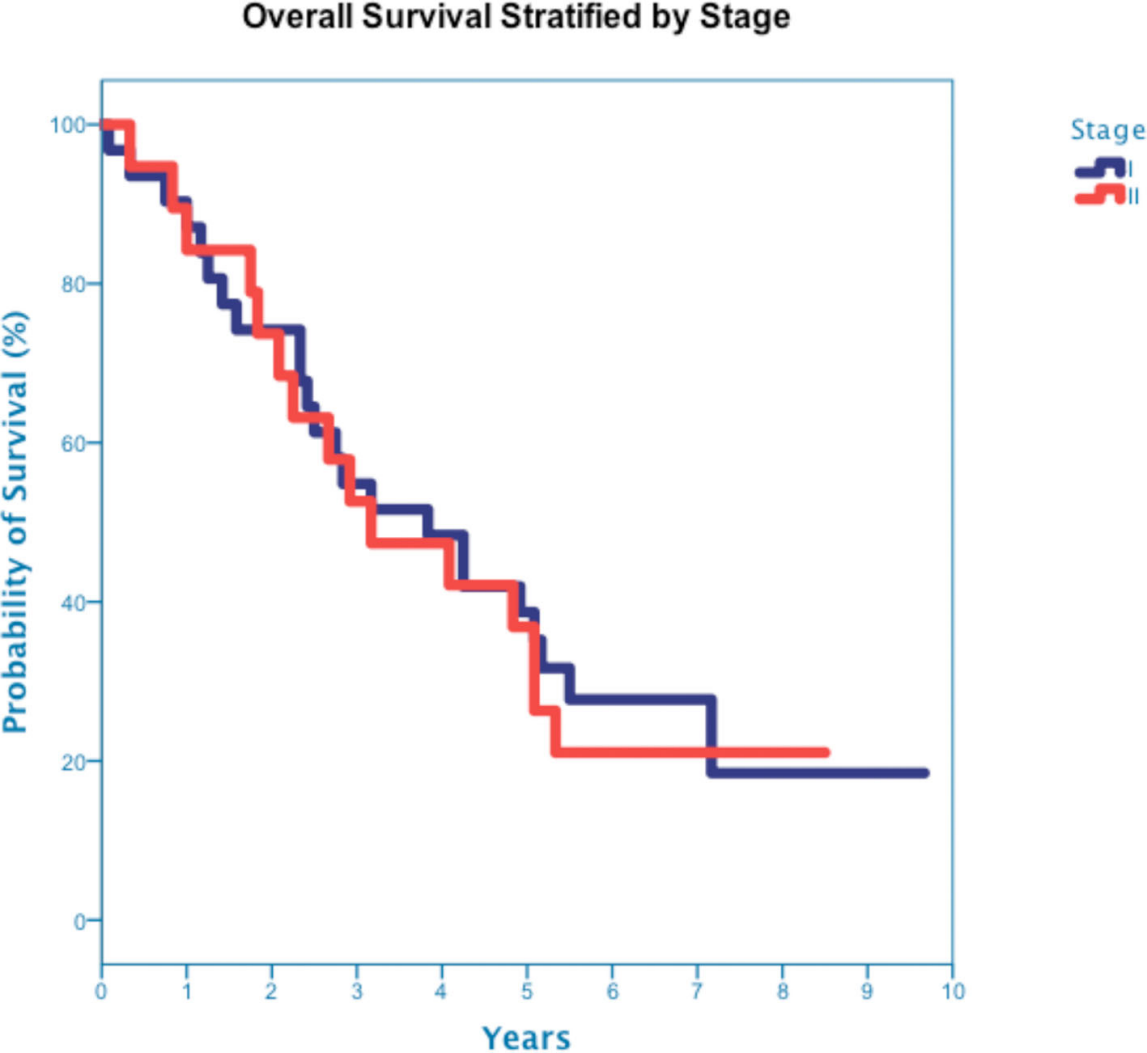
Overall Survival stratified by Stage I and stage II disease (p-value =0.86).

**Figure 5:**
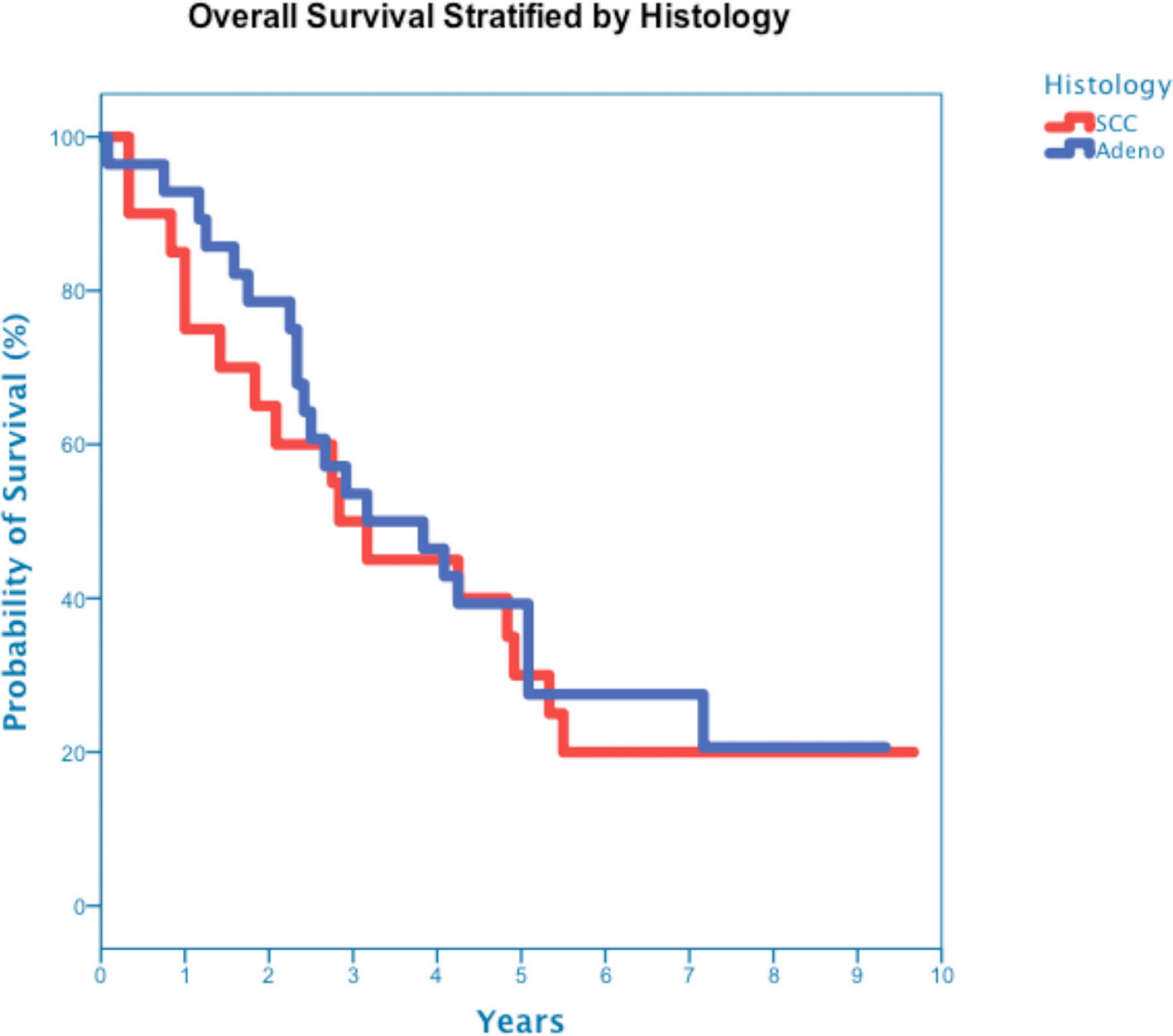
Overall Survival stratified by Histology squamous cell carcinoma and adenocarcinoma (p-value = 0.68).

**Figure 6:**
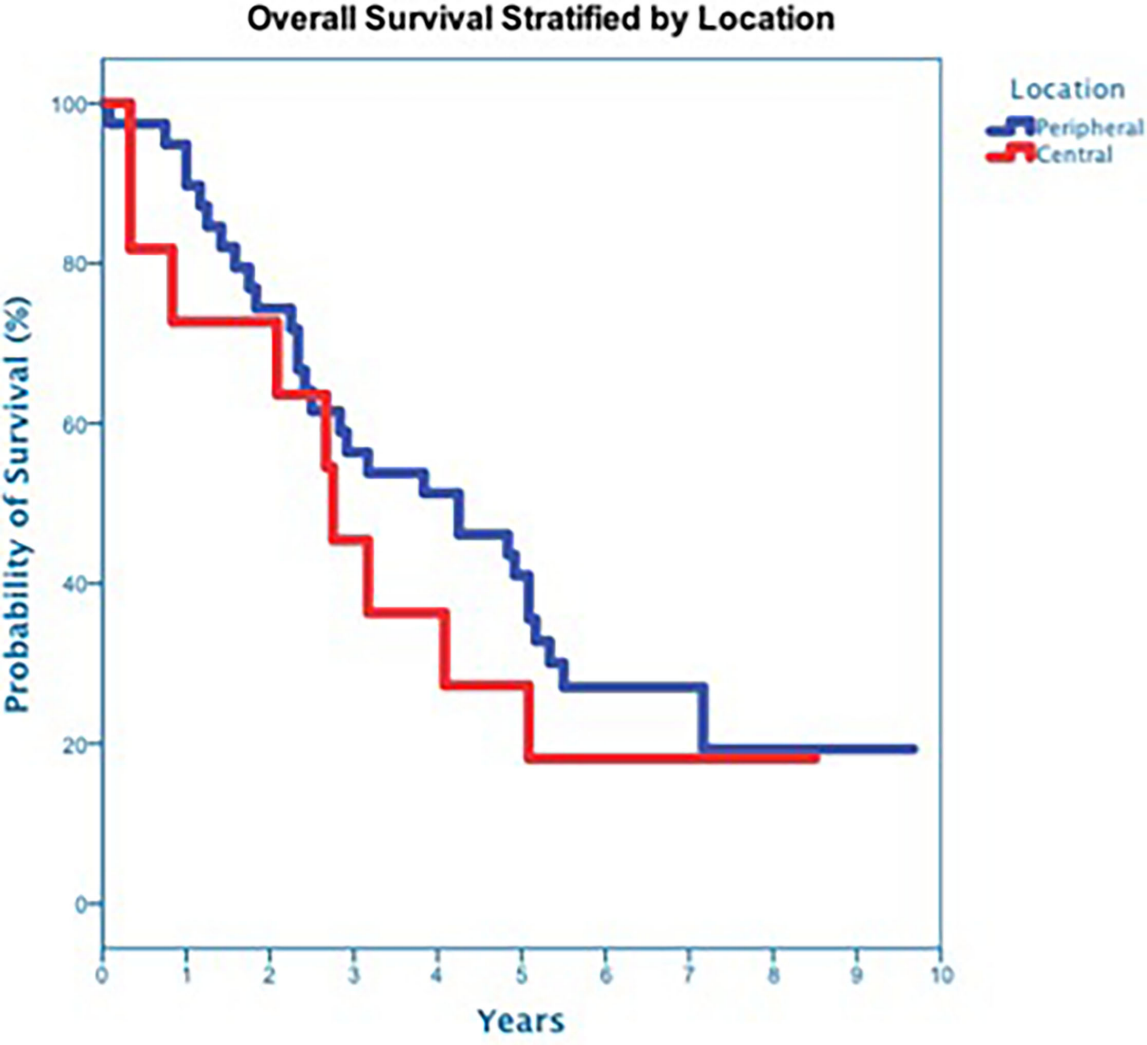
Overall Survival stratified by Location (p-value = 0.46).

At a median follow up of 42 months, 40 deaths had occurred, and 3-year OS was 50% (**Figure 3**). Three-year OS did not differ statistically between stage I and stage II disease (51% *vs*. 47%; p=0.86), squamous cell carcinoma and adenocarcinoma (45% *vs*. 50%; p=0.68) or peripheral and central tumors (56% *vs*. 45%; p=0.46) (**Figures 4**–**6**). Eighteen deaths were attributed to metastatic NSCLC (45%), 14 deaths were the result of comorbid illness (35%), four deaths where the result of treatment (10%), and three deaths were attributed to second primaries (7.5%). The lethal second primaries included pancreas, colon and anal cancer. Three-year LC, RC, DMFS and DFS results was 90%, 90%, 82% and 65%, respectively.

### Bronchoscopy

**Figure 7:**
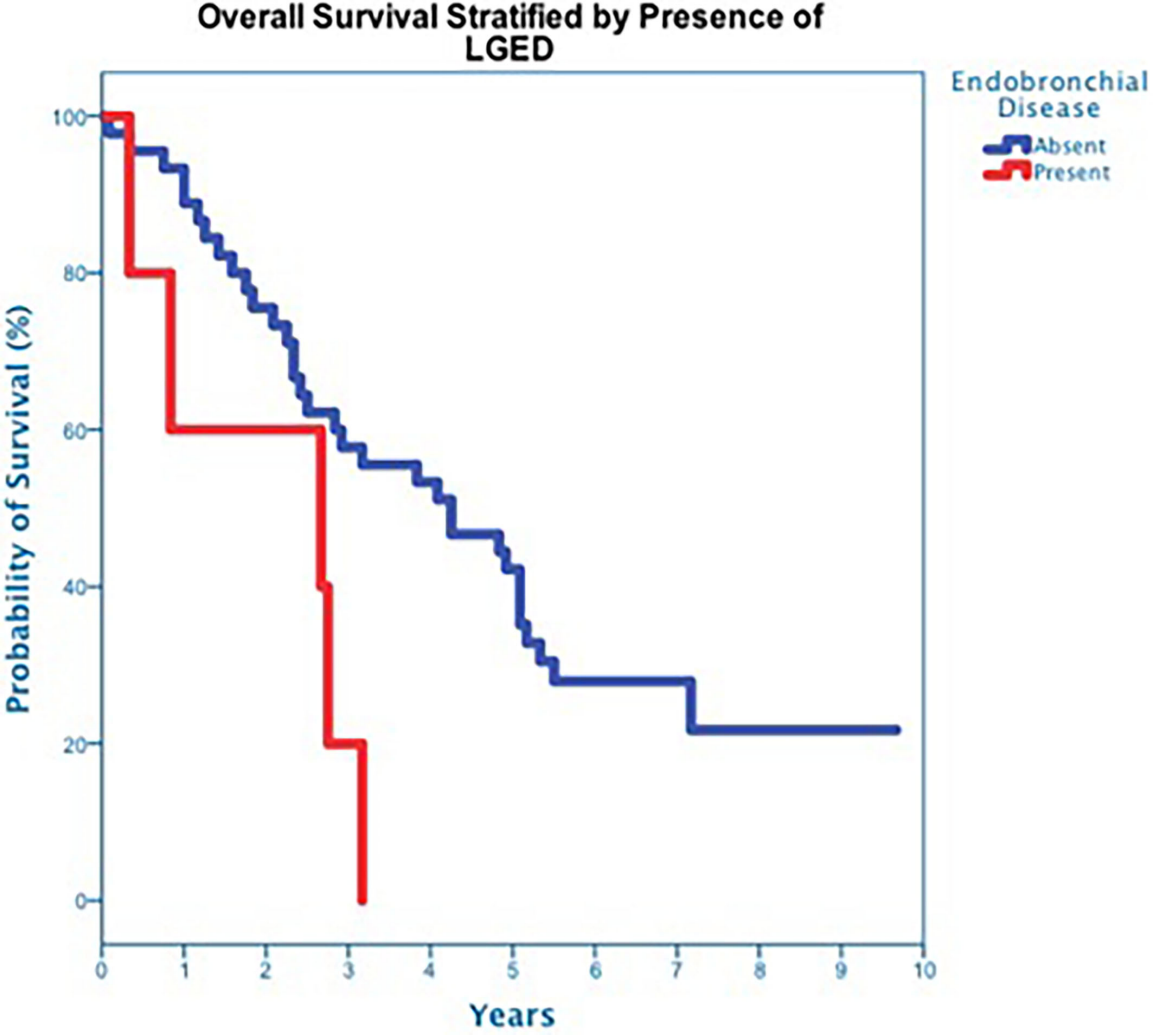
Overall Survival stratified by presence of lobar endobronchial disease.

The majority of patients (82%), including all eleven central tumor patients, underwent staging bronchoscopy. There was no evidence of tumor invasion into the mainstem bronchus or trachea in any patient who underwent bronchoscopy. Five tumors, all of which were centrally located, were found to have LGED (45% of the central tumors) and involved the RUL (n=2), RLL (n=1), LUL (n=1) and LLL (n=1). Tumors with LGED were predominately squamous cell carcinoma (80%) and clinical stage II (60%) (**Table 4**). Three-year OS for tumors with LGED was quite poor at 20% (**Figure 7**). Cox regression analysis identified LGED as an independent predictor of OS while controlling for age, stage and location (OR:4.536, *p-value*=0.038). Despite our relatively low dose and tight margins, treatment likely contributed to the death of four patients with centrally located squamous cell carcinoma (**Table 4**). In addition, LGED was an independent predictor for grade 5 pulmonary toxicity (n=4, p=0.007). Specifically, 3 of 5 patients with LGED likely developed fatal radiation-induced bronchial stricture. The median maximum dose to the lobar bronchus in these 3 patients was 67.3 Gy (range 62.3-68.0 Gy) (**Table 4**). A fourth patient, having undergone left pneumonectomy for tuberculosis in the distant past, likely died of radiation pneumonitis approximately 5 months following the treatment of a right central squamous cell carcinoma surrounding a lobar bronchus but without LGED. The maximum dose to the lobar bronchus in this patient was 63 Gy (**Table 4**). Two central lung tumors that developed pathologically confirmed local failures in our study did so despite having received high maximum doses to the lobar bronchus of 65 Gy (**Table 4**).

**Table 4:** Centeral tumor characteristics and SBRT outcomes.

Patient ID	Histology	LGED	T-Stage	Max Dose to Lobar Bronchi	Grade 5 Toxicity	Local Failure	Vital status
Patient 1	SCC	No	T3	59.5	No	No	Alive
Patient 2	SCC	Yes	T1b	62.3	Yes	No	Dead
Patient 3	SCC	No	T3	63	Yes	No	Dead
Patient 4	SCC	No	T2b	57	No	Yes	Dead
Patient 5	SCC	Yes	T2a	65	No	Yes	Dead
Patient 6	SCC	Yes	T3	68	Yes	No	Dead
Patient 7	Adeno	No	T2b	33.7	No	No	Dead
Patient 8	Adeno	No	T2b	65	No	Yes	Dead
Patient 9	SCC	No	T2b	58.7	No	No	Alive
Patient 10	SCC	Yes	T2b	67.3	Yes	No	Dead
Patient 11	Adeno	Yes	T2b	57	No	No	Dead

## Discussion

In the earliest SBRT prospective phase II trial of its kind, Timmerman et al. treated 70 patients with inoperable stage I NSCLC between January 2002 and July 2004 with the near maximum tolerated doses of 60 and 66 Gy in 3 fractions delivered to the 80% isodose line for T1 (34 patients) and T2 (36 patients) tumors (BED_10_ > 150) using a conventional SBRT technique (1–4). At a median follow-up of 50.2 months, 3 year LC and OS where 88.1% and 42.7% (4). There was no significant survival difference between the 22 patients with central tumors and the 48 patients with peripheral tumors (4). However, the aggressive treatment delivered at near maximum tolerated dose may have contributed to the death of five patients with predominantly central lung tumors (7%) (3, 4).

In 2010, we initiated a single institution non-randomized protocol for medically inoperable patients with ES-NSCLC. In this era, SBRT was the only feasible radical treatment option available for medically inoperable central ES-NSCLC patients and was therefore delivered despite the risk of life-threatening toxicity. Our goal was to reliably eradicate tumors with the minimum margin and dose necessary in order to curtail such life-threatening toxicity. Ultimately, a group of 50 consecutive patients with central (11) and peripheral (39) tumors were treated over a 5-year period extending from December 2010 until December 2015 and followed for a minimum of 5 years or until death. At a median follow-up of 42 months, 3-year LC and OS were similar to the Indiana University experience at 90% and 50% (4). Three-year OS did not differ statistically between peripheral and central tumors as seen in the Indiana University experience (4). However, despite our relatively tight margins and low dose, treatment may have contributed to the death of four patients (8%) also surprisingly similar to the Indiana University experience (4).

Pretreatment bronchoscopic evaluation of the proximal airway in our patient population of ES-NSCLC patients has been routine since 2005 when our institution published the first manuscript detailing a procedure for placing fiducials for lung SBRT using bronchoscopy (9). This procedure was developed in order to reduce the significant risk of iatrogenic pneumothorax that had been noted with CT-guided fiducial placement (7, 9–12). Early clinical outcomes research also completed by our team evaluating the role of SBRT in the treatment of central lung metastases identified gross endobronchial disease (GED) at the time of staging bronchoscopy as a potential predictor of poor overall survival and SBRT related death (21, 22). In the present ES-NSCLC study, no mainstem bronchus GED was identified. However, we have confirmed pretreatment LGED is a significant negative prognostic factor. This could represent a hitherto unappreciated pathophysiological basis for the increased risk associated with the SBRT of central ES-NSCLC.

Here we identify LGED as a significant predictor of poor overall survival in inoperable ES-NSCLC patients treated with SBRT. Also, we propose the relatively high rates of severe treatment-related toxicity reported in patients with central ES-NSCLC undergoing SBRT may be due in part to LGED damaging the lobar bronchi prior to SBRT. However, a critical issue concerning the validity of our observations does exist. It is possible that the severe toxicity seen in our study is the result of radiation damage alone and not the result of tumor destruction of the lobar bronchi prior to radiation therapy. It is not possible to distinguish these two distinct causes of death based on clinical presentation and radiographic evaluation alone. Autopsies were not completed on these patients. Unfortunately, tumors with LGED have directly invaded the lobar bronchus and therefore delivering radical doses to this structure is unavoidable.

The advent of SBRT for the radical treatment of early stage non-small cell lung cancer has fundamentally changed the way we evaluate organs at risk in the thorax. For decades, radiation doses to the lobar bronchus have been ignored, but in the SBRT era this organ can no longer be taken for granted. The proximal bronchial tree is a serial organ, thus damage to any segment, whether from tumor or radiation, may lead to profound downstream effects and ultimately high-grade toxicities. It is also similar to other serial organs such as the spinal cord and intestinal tract, in that once the functional ability of a segment of the organ has been sufficiently infringed upon by the tumor, no dose or manner of radiation is able to functionally restore that organ.

We delivered the minimal effective dose using the tightest margins available and therefore did not use lobar bronchus dose constraints. The median maximum dose to the lobar bronchus for patients with LGED was high at 65 Gy (range 57 - 68 Gy). It may be possible that lobar bronchus dose constraints could have limited treatment related deaths and enhanced overall survival, however we do not believe this to be the sole factor promoting grade 5 toxicity in this cohort. There were no grade 5 pulmonary toxicities seen in patients with lobar bronchus maximum point dose less than 62.3 Gy. Two central tumor study patients, one of which had LGED, developed pathologically confirmed isolated local failures despite having received high maximum point doses to the lobar bronchus of 65 Gy (**Table 4**). These patients ultimately died of metastatic disease. In our opinion, limiting doses to the lobar bronchus below what was delivered here would only add to the already high local failure rate seen in this study for central tumors and is not a viable solution (**Table 4**). At a minimum our research, not unlike the Indiana University experience, suggests that the therapeutic window for SBRT when tumors closely approach or grossly invade the lobar bronchus is narrow and therefore alternative treatment approaches may be required to achieve satisfactory results in this patient population. Alternative fractionations or the use of chemotherapy may impact both the disease outcome and risk of toxicity in this patient population, however, our research suggests that as the anatomical separation between the tumor and the lobar bronchus narrows, so does the therapeutic window for thoracic SBRT. Future studies are needed to further elucidate the relationship between LGED and risk of high-grade toxicity.

## Conclusion

Central location of ES-NSCLC is a well-established predictor for SBRT-related toxicity. Here we identify LGED as a significant predictor of poor overall survival and grade 5 pulmonary toxicity. The relatively high rates of severe treatment-related toxicity reported in patients with central ES-NSCLC may be due in part to LGED. Future prospective central ES-NSCLC clinical trials should require staging bronchoscopy to identify LGED and further assess its clinical significance.

## Data Availability Statement

The raw data supporting the conclusions of this article will be made available by the authors, without undue reservation.

## Ethics Statement

The studies involving human participants were reviewed and approved by Georgetown University IRB. Written informed consent for participation was not required for this study in accordance with the national legislation and the institutional requirements.

## Author Contributions

BC and NA conceived of the project. NA conducted data analysis. NA, CH, and TO’C collected data. All authors contributed to the article and approved the submitted version.

## Conflict of Interest

SC receives research funding from Accuray Inc.

The remaining authors declare that the research was conducted in the absence of any commercial or financial relationships that could be construed as a potential conflict of interest.

## Publisher’s Note

All claims expressed in this article are solely those of the authors and do not necessarily represent those of their affiliated organizations, or those of the publisher, the editors and the reviewers. Any product that may be evaluated in this article, or claim that may be made by its manufacturer, is not guaranteed or endorsed by the publisher.
